# Managing allorejection in off-the-shelf CAR-engineered cell therapies

**DOI:** 10.1016/j.ymthe.2024.11.035

**Published:** 2024-11-26

**Authors:** Yan-Ruide Li, Ying Fang, Siyue Niu, Yuning Chen, Zibai Lyu, Lili Yang

**Affiliations:** 1 Department of Microbiology, Immunology & Molecular Genetics, University of California, Los Angeles, Los Angeles, CA 90095, USA; 2 Department of Bioengineering, University of California, Los Angeles, Los Angeles, CA 90095, USA; 3 Molecular Biology Institute, University of California, Los Angeles, Los Angeles, CA 90095, USA; 4 Eli and Edythe Broad Center of Regenerative Medicine and Stem Cell Research, University of California, Los Angeles, Los Angeles, CA 90095, USA; 5 Jonsson Comprehensive Cancer Center, David Geffen School of Medicine, University of California, Los Angeles, Los Angeles, CA 90095, USA; 6 Parker Institute for Cancer Immunotherapy, University of California, Los Angeles, Los Angeles, CA 90095, USA

**Keywords:** allorejection, off-the-shelf cell therapy, allogeneic cell therapy, chimeric antigen receptor-engineered T cells, CAR-T cells, genetic engineering, natural killer cells, NK cells, human leukocyte antigen, HLA, beta-2-microglobulin, B2M, class II major histocompatibility complex transactivator, CIITA

## Abstract

Chimeric antigen receptor (CAR)-engineered T (CAR-T) cell therapy has revolutionized the treatment of various diseases, including cancers and autoimmune disorders. However, all US Food and Drug Administration (FDA)-approved CAR-T cell therapies are autologous, and their widespread clinical application is limited by several challenges, such as complex individualized manufacturing, high costs, and the need for patient-specific selection. Allogeneic off-the-shelf CAR-engineered cell therapy offers promising potential due to its immediate availability, consistent quality, potency, and scalability in manufacturing. Nonetheless, significant challenges, including the risks of graft-versus-host disease (GvHD) and host-cell-mediated allorejection, must be addressed. Strategies such as knocking out endogenous T cell receptors (TCRs) or using alternative therapeutic cells with low GvHD risk have shown promise in clinical trials aimed at reducing GvHD. However, mitigating allorejection remains critical for ensuring the long-term sustainability and efficacy of off-the-shelf cell products. In this review, we discuss the immunological basis of allorejection in CAR-engineered therapies and explore various strategies to overcome this challenge. We also highlight key insights from recent clinical trials, particularly related to the sustainability and immunogenicity of allogeneic CAR-engineered cell products, and address manufacturing considerations aimed at minimizing allorejection and optimizing the efficacy of this emerging therapeutic approach.

## Graphical abstract

**Figure:**
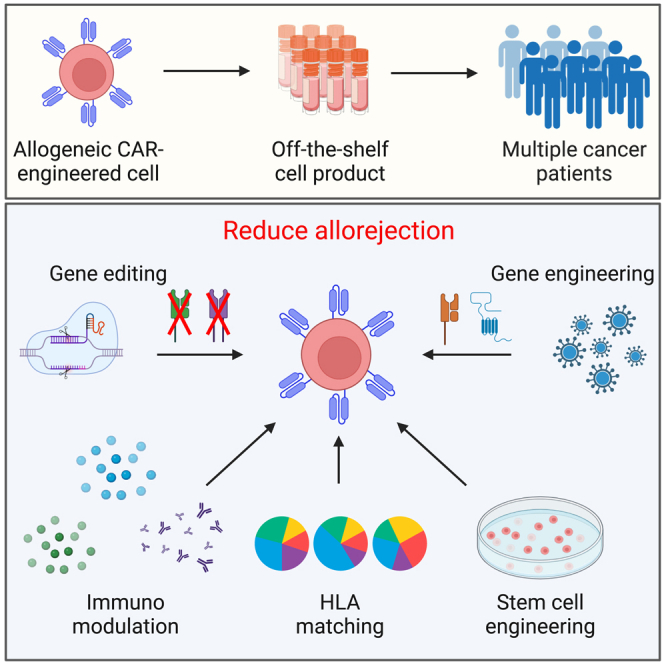

## Teaser

Li and colleagues emphasize the critical challenge of managing host-cell-mediated allorejection in off-the-shelf CAR therapies. They review current preclinical and clinical strategies to mitigate allorejection, highlight insights from recent trials on cell product persistence and immunogenicity, and discuss future manufacturing considerations to improve efficacy and reduce allorejection.

## Introduction

Autologous chimeric antigen receptor (CAR)-engineered T (CAR-T) cell therapy, which is generated from a patient’s own T cells, has significantly transformed the treatment paradigm for hematologic malignancies and solid tumors.^1^^,^^2^^,^^3^^,^^4^ Despite its success, widespread clinical application is hindered by several limitations, including logistical complexities in the supply chain, variability in patient T cell health, delays due to individualized manufacturing processes, and limited manufacturing capacity.^1^ To address these challenges, there is a growing interest in developing "off-the-shelf," standardized treatments. Allogeneic T cell and natural killer (NK) cell therapies, especially those engineered with CARs, offer considerable advantages in this regard.^5^^,^^6^^,^^7^^,^^8^ These therapies utilize lymphocytes derived from healthy donor peripheral blood or placental cord blood, providing a readily available source of functional immune cells. Additionally, lymphocytes differentiated from genetically engineered stem cells, such as induced pluripotent stem cells (iPSCs) or hematopoietic stem cells (HSCs), are being explored as alternative sources.^9^^,^^10^^,^^11^^,^^12^

Allogeneic cell therapy presents several advantages over autologous approaches, positioning it as a promising strategy in cell-based cancer therapy.^13^ Primarily, it offers immediate availability of treatment, as donor cells can be pre-collected, processed, and stored for off-the-shelf use, significantly reducing the time to treatment and eliminating the need for bridging therapies. The use of healthy donor cells ensures consistent quality and potency, with cells exhibiting better phenotypic characteristics, lower exhaustion marker expression, and enhanced long-term cytotoxic activity compared to cells from immunocompromised patients. This approach allows for large-scale manufacturing from a single donor batch, improving scalability and manufacturing efficiency and reducing costs, thereby increasing accessibility for a broader patient population.^13^^,^^14^ Furthermore, donor cells can be selected for desirable traits or genetically modified to enhance efficacy and reduce immunogenicity, overcoming limitations associated with autologous cells from patients with genetic disorders or advanced diseases.^9^^,^^15^ The standardization of therapy using allogeneic cells reduces variability in therapeutic outcomes linked to patient-specific factors, simplifies supply chain logistics, and expands treatment availability beyond specialized centers to any facility equipped to administer cell infusions.^13^^,^^14^ Collectively, these benefits reduce the procedural burden on cancer patients, expedite treatment initiation, and have the potential to improve clinical outcomes through enhanced therapeutic efficacy and broader accessibility.

While off-the-shelf CAR-engineered cell products offer significant benefits, key obstacles must be addressed to make allogeneic cell therapy viable for patients. The primary concern is graft-versus-host disease (GvHD), a serious condition that can occur when infused CAR-engineered cells from an unmatched donor attack the host’s tissues.^16^^,^^17^^,^^18^^,^^19^ This happens because the donor T cell receptor (TCR) complex recognizes the host’s peptide-major histocompatibility complex (MHC) as foreign. To prevent GvHD, several strategies are being explored. One approach is using donor cells fully matched for human leukocyte antigen (HLA) alleles, but finding a perfect HLA match is rare and impractical. Alternative methods include genetically knocking out or suppressing TCR genes in donor cells to eliminate their ability to react against the host’s MHC molecules.^20^^,^^21^^,^^22^^,^^23^^,^^24^ Using cells with known TCR specificity that does not target host antigens, or employing cell types that naturally lack the TCRαβ complex, such as NK or innate T cells, can also reduce the risk.^25^^,^^26^^,^^27^^,^^28^^,^^29^ These strategies aim to harness allogeneic cell therapy’s potential while overcoming immunological challenges posed by donor-recipient mismatches.

However, the host immune system can recognize the allogeneic graft as foreign, triggering rejection and limiting therapeutic efficacy.^7^^,^^9^^,^^30^ To address this challenge, developers are implementing various strategies to circumvent host rejection of allogeneic cell therapies. In this review, we specifically focus on the allorejection of CAR-engineered therapies, discussing the immunological basis of allorejection and various strategies to mitigate it. We also examine the clinical application of allogeneic cell therapy, emphasizing lessons learned from clinical trials, particularly regarding the sustainability and immunogenicity of allogeneic CAR-engineered cell products. Furthermore, we highlight manufacturing considerations for allogeneic cell therapy aimed at reducing allorejection and enhancing the overall efficacy of this promising therapeutic approach.

## Immunological basis of allorejection for cell-based therapy

**Figure 1:**
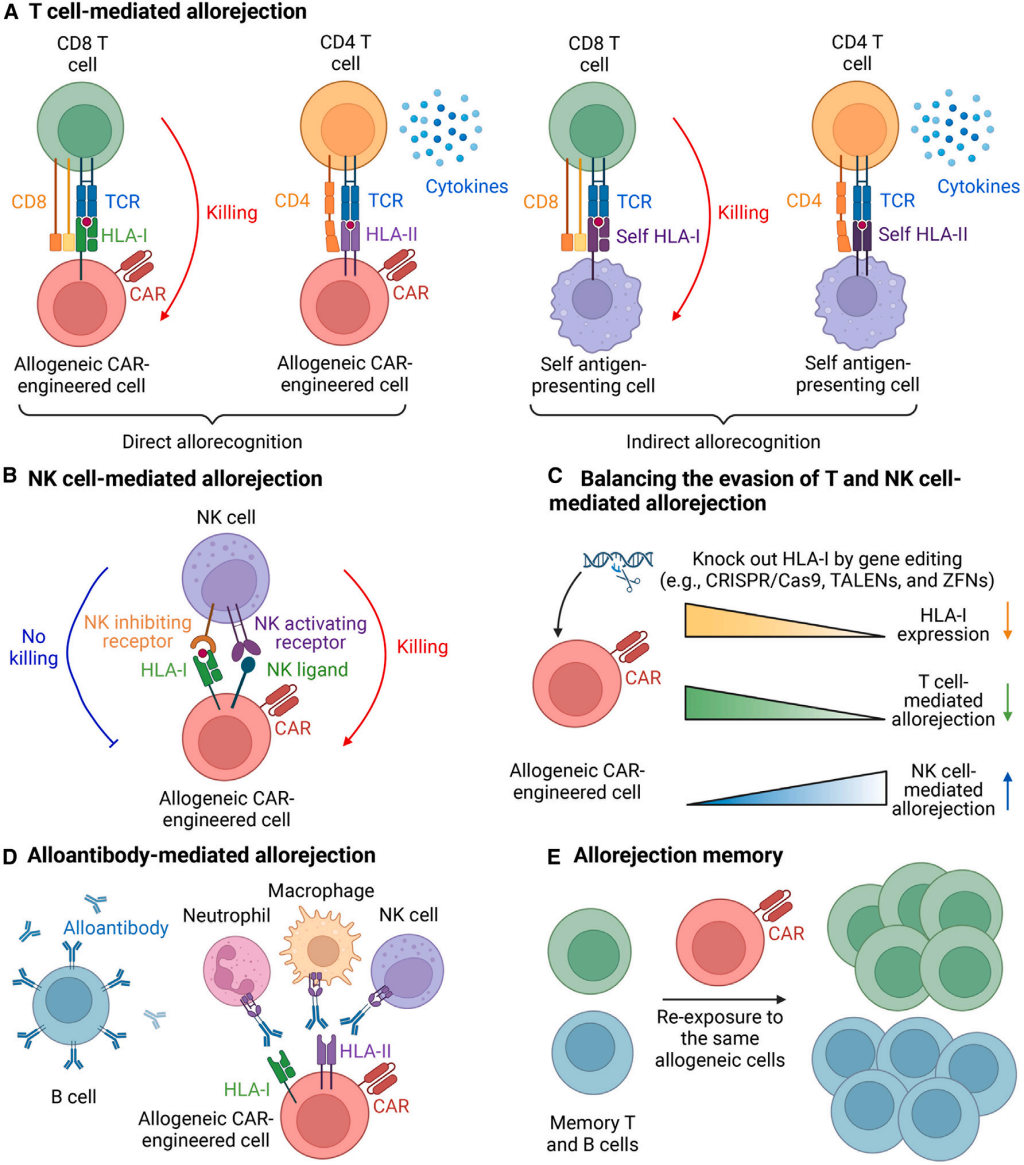
Immunological basis of allorejection for off-the-shelf CAR-engineered cells (A) T cell-mediated allorejection. CD4 and CD8 T cells mediate direct and indirect allorecognition in response to allogeneic cells. In direct allorecognition, CD4 T cells recognize HLA-II molecules, while CD8 T cells recognize HLA-I molecules on the surface of allogeneic cells. CD4 T cells primarily facilitate the recruitment of additional immune effectors, whereas CD8 T cells directly mediate the killing of the allogeneic cells. In indirect allorecognition, donor antigens are processed by host APCs and presented as peptide fragments bound to self-MHC molecules. Host CD4 or CD8 T cells recognize these peptide-MHC complexes, triggering an immune response against the allogeneic cells. (B) Host NK cells can trigger allorejection through a dual-trigger mechanism. This mechanism involves two aspects: (1) missing self, which occurs when there is a lack of matching HLA-I molecules on allogeneic cells, leading to the blockade of inhibitory signals mediated by NK inhibitory receptors (e.g., KIRs); (2) stress signals, which involve the upregulation of stress molecules on allogeneic cells, resulting in the activation of NK-activating receptors (e.g., NKG2D). (C) Gene-engineering strategies have been employed to knock out HLA-I molecules on allogeneic cells to prevent host T cell-mediated allorejection. However, knocking out HLA-I increases the risk of NK cell-mediated allorejection, as the absence of matching HLA-I molecules triggers NK cell activation through the missing-self mechanism. TALENs, transcription activator-like effector nucleases; ZFNs, zinc-finger nucleases. (D) Allo-specific antibodies (alloantibodies) produced by host B cells contribute to the rejection of allogeneic cells by primarily recognizing foreign HLA molecules, although they may also target other polymorphic surface proteins. These antibodies mediate rejection via complement fixation, antibody-dependent cellular cytotoxicity (ADCC), and antibody-dependent cellular phagocytosis (ADCP), involving immune cells such as NK cells, neutrophils, monocytes, and macrophages, which collaborate to eliminate the targeted allogeneic cells. (E) Host T and B cells are capable of developing immunologic memory. Upon re-exposure to the same allograft, such as during subsequent treatments, these memory cells undergo rapid expansion, resulting in accelerated clearance of the reinfused allogeneic cells.

Several effector responses mediate allorejection, and these must be addressed to prevent the rejection of allogeneic cell therapies. The two primary mechanisms of allorejection in allogeneic cell therapy are mediated by host T cells and NK cells (Figure 1), both of which pose significant challenges to the success of transplantation and adoptive cell therapies.

Host T cell-mediated allorejection is a critical immune response in which the recipient’s T cells recognize and attack transplanted allogeneic cells.^31^ This response is initiated when the immune system perceives donor cells as foreign, primarily due to differences in HLA molecules between the donor and recipient. The recognition of allogeneic cells by host T cells can occur via direct allorecognition or indirect allorecognition (Figure 1A).^31^

In the T cell-mediated direct allorecognition, the polymorphic differences between the HLA class I and class II molecules of the donor and recipient lead to the activation of host CD8^+^ and CD4^+^ T cells, respectively.^32^^,^^33^ Host T cells directly interact with intact alloantigen-MHC complexes presented on the surface of donor antigen-presenting cells (APCs). This direct recognition can trigger a potent immune response, resulting in the activation of cytotoxic CD8^+^ T cells, which can directly kill donor graft cells. This pathway is particularly important in acute allorejection, where rapid immune activation can lead to graft failure.^31^^,^^34^

In the T cell-mediated indirect allorecognition, donor antigens are processed by recipient APCs and presented as peptide fragments bound to self-MHC molecules. Host CD4^+^ T cells recognize these peptide-MHC complexes, which initiates an immune response.^33^^,^^34^ Unlike direct allorecognition, this pathway is more likely to contribute to chronic rejection, as it involves a slower, more prolonged immune response through the activation of helper T cells, which can facilitate the recruitment of additional immune effectors, such as B cells and macrophages, further amplifying the rejection process.^34^

Host NK cells contribute to allorejection through a dual-trigger mechanism that involves both the recognition of "missing-self" signals and the detection of "stress signals" on allogeneic cells (Figure 1B).^10^^,^^11^ This mechanism can undermine the efficacy of allogeneic cell therapies, particularly when strategies are employed to evade T cell-mediated rejection. On the one hand, NK cells are programmed to detect the absence or downregulation of self HLA-I molecules on target cells. In the case of allogeneic cells, if these cells lack or present non-matching HLA-I molecules, NK cells fail to receive inhibitory signals typically mediated by killer cell immunoglobulin-like receptors (KIRs), leading to NK cell activation. This missing-self recognition prompts NK cells to attack and eliminate the allogeneic cells. On the other hand, NK cells can also be activated by the upregulation of stress-induced ligands on the surface of allogeneic cells. These stress molecules, often upregulated due to cellular distress or genetic modifications in allogeneic therapies, engage activating NK receptors (NKRs) such as NKG2D. The binding of stress ligands to these activating receptors further amplifies NK cell-mediated cytotoxicity.^10^^,^^11^^,^^35^

Together, the missing-self and stress-signal mechanisms create a potent dual-trigger system by which host NK cells can recognize and destroy allogeneic cells. This presents a significant challenge in the development of off-the-shelf CAR-engineered therapies, especially when HLA downregulation is used to evade host T cell recognition. To mitigate NK cell-mediated allorejection, strategies such as expressing non-polymorphic HLA molecules (e.g., HLA-E and HLA-G) or modifying NK ligands on allogeneic cells are being explored (e.g., reducing expression of NK ligands).^10^^,^^11^^,^^36^^,^^37^ These approaches aim to balance the evasion of both T and NK cell responses while maintaining the therapeutic efficacy of the engineered cells (Figure 1C).^38^ Indeed, a recent study on HLA-I knockdown in allogeneic CAR-T cell products indicates that reducing HLA-I expression on CAR-T cells enhances their resistance to host T cell-mediated allorejection but increases their susceptibility to NK cell-mediated rejection.^39^ The study suggests that an optimal HLA-I knockdown, maintaining HLA-I expression levels at 20%–50% of that in conventional CAR-T cells, provides the best balance between resistance to both T cell- and NK cell-mediated allorejection.^39^ This finding highlights the importance of fine-tuning HLA-I expression to maximize the persistence and efficacy of allogeneic CAR-T cell therapies.

In addition to T and NK cell responses, allo-specific antibodies produced by host B cells can contribute to the rejection of allogeneic cells.^40^^,^^41^ These antibodies primarily recognize foreign HLA molecules on graft cells but can also target other polymorphic surface molecules. Once bound to the graft cells, these antibodies can mediate rejection through several mechanisms, including complement fixation, antibody-dependent cellular cytotoxicity (ADCC), and antibody-dependent cellular phagocytosis (ADCP).^40^ These processes involve various immune cells, such as NK cells, neutrophils, monocytes, and macrophages, which work together to eliminate the targeted cells (Figure 1D).

Furthermore, both recipient T cells and B cells are capable of developing immunologic memory. Upon re-exposure to the same allograft, such as during subsequent treatments, these memory cells rapidly expand, leading to accelerated clearance of the reinfused allogeneic cells (Figure 1E).^42^ This immunologic memory presents a significant challenge to the long-term success of allogeneic therapies, as it can result in reduced efficacy upon repeated administration.^42^ Understanding and mitigating the formation of allo-specific antibodies and memory immune responses are critical for improving the persistence and effectiveness of allogeneic cell therapies.

## *In vitro* and *in vivo* assays to evaluate allorejection

**Figure 2:**
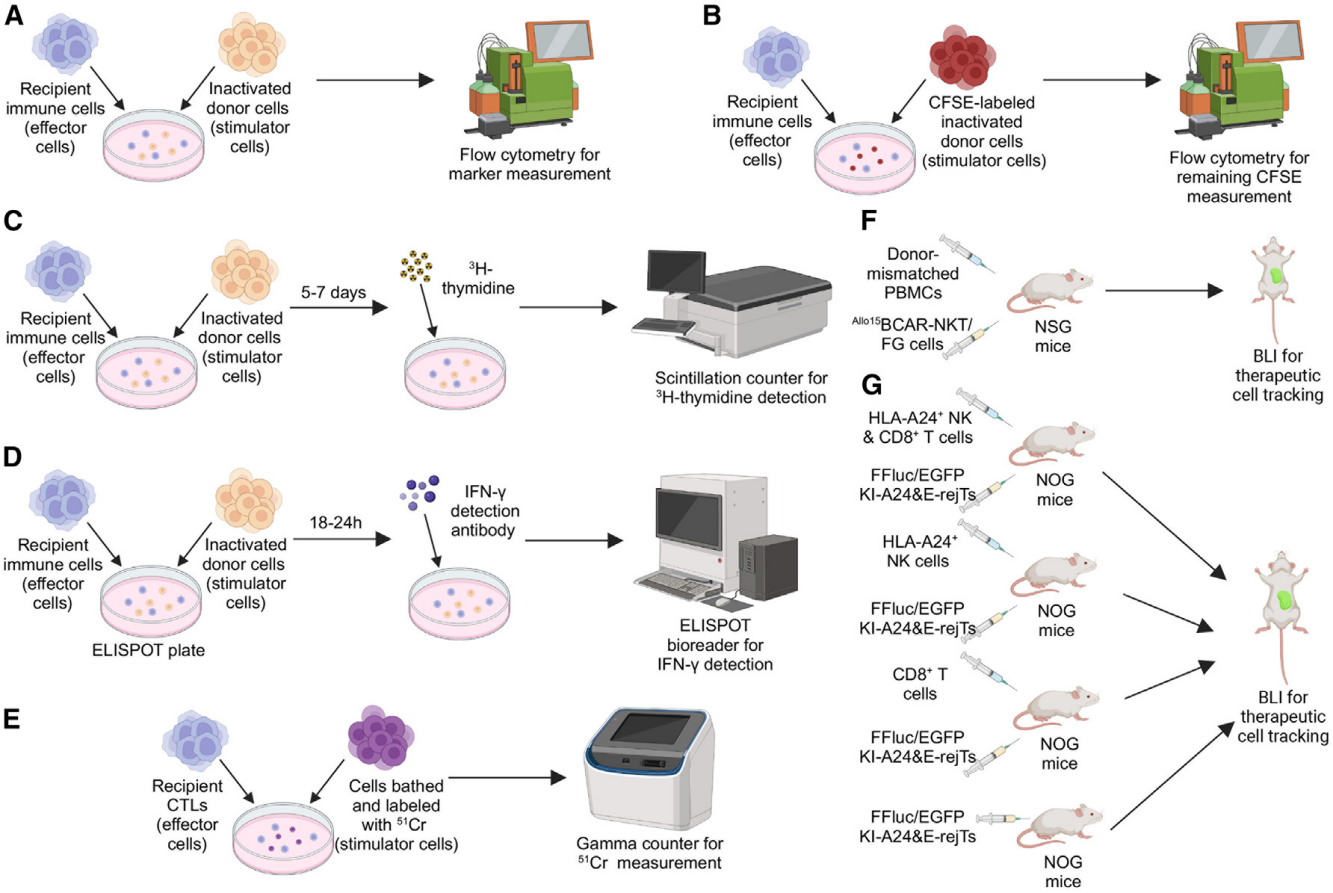
*In vitro* and *in vivo* assays assessing allorejection A range of assays has been employed to assess host-cell-mediated allorejection of grafted cells, including flow cytometry in mixed lymphocyte reaction (MLR) assays (A), carboxyfluorescein succinimidyl ester (CFSE) staining (B), ^3^H-thymidine incorporation (C), IFN-γ detection through antibody labeling (D), and ^51^Cr release (E). Humanized mouse models, such as NSG mice engrafted with human PBMCs and ^Allo15^BCAR-NKT cells (F), and NOG mice with human HLA-A24^+^ NK and/or CD8^+^ T cells and KI-A24&E-rejTs (G), can also be used for *in vivo* allorejection evaluations.^10^^,^^43^

To evaluate the host-cell-mediated allorejection of allogeneic CAR-engineered cell therapies, various *in vitro* and *in vivo* assays have been developed to model and measure the immune response to the allogeneic cells (Figure 2).

One commonly used *in vitro* assay for measuring allorejection is the mixed lymphocyte reaction (MLR) assay. Culturing lymphocytes from genetically distinct individuals of the same species (stimulator cells and effector cells) together leads to blast transformation, making the MLR a valuable tool for clinically evaluating a recipient’s immune response to donor cells.^10^^,^^11^^,^^44^ In one-way assay testing allograft rejection, stimulator cells are typically inactivated through gamma irradiation, ensuring that only the recipient’s cells can react.^44^ Flow cytometry and ELISA are commonly utilized to detect a range of markers related to allorejection on co-cultured products, such as T cell activation markers CD25, CD69, and CD137, as well as interferon (IFN)-γ.^45^

Moreover, to evaluate the immune response, stimulator cells can be labeled with antibodies such as carboxyfluorescein diacetate succinimidyl ester (CFSE), and flow cytometry is included to obtain results. For example, in a study focusing on the antitumor effect of HLA-A^−^/B^−^/TRAC^−^ novel CD19-targeting CAR-T (nU-CAR-T19) cells against B cell malignancies, nU-CAR-T19 and wild-type (WT) T or U-CAR-T19 cells were used as stimulator cells, whereas allogeneic PBMCs, T cells, or NK cells were employed as effector cells.^45^ The inactivated nU-CAR-T19 and WT T or B2M^−^/TRAC^−^ universal CD19-targeting CAR-T (U-CAR-T19) cells were irradiated, labeled with CFSE, and co-cultured with the effector cells. Following the MLR culture, the harvested cells were analyzed using flow cytometry to measure the total number of remaining CFSE-positive cells that were not eliminated by the effector cells, demonstrating the immune response of effector cells and the safety of the allogeneic therapeutic cells.^45^ Besides CFSE, the incorporation of 3H-thymidine into the co-cultured product is used to detect alloreactive responses. As 3H-thymidine incorporates into the DNA of the proliferating effector cells, it provides a quantitative measure of allorejection by indicating the alloreactive proliferation of recipient cells.^46^ Furthermore, IFN-γ enzyme linked immunosorbent spot (ELISPOT) assay is used to detect the presence of circulating memory/effector T cells. For this method, the MLR process is conducted within the ELISPOT plate. After 18–24 h, the IFN-γ detection antibody is added to the plate for the evaluation of alloreactive primed T cells.^46^^,^^47^

Another *in vitro* assay testing allograft rejection response is the chromium-51 (^51^Cr) release assay. Target cells are first bathed and labeled with chromium and then mixed with allogeneic cytotoxic T lymphocytes (CTLs). Similar to the MLR assay, if target cells express identical or cross-reactive antigens to effector cells, these labeled cells will be lysed, releasing chromium into the medium. Then the allorejection response is assessed by measuring the accumulated chromium in the medium and comparing it to the spontaneous release amount.^48^ To test the hypoimmunogenic characteristic of HLA class I-edited functionally rejuvenated human papilloma virus-specific CTLs that feature dual integration of HLA-A24 and HLA-E without other class I molecule expression (KI-A24&E-rejTs) generated from iPSCs, Furukawa et al. co-cultured KI-A24&E-rejTs with allogeneic NK cells from HLA-A24^+^ healthy donors. The lower chromium level within the mixture of KI-A24&E-rejTs and NK cells proves the therapeutic cells’ suppression on NK cells.^43^

In addition to *in vitro* assays, *in vivo* assays are developed in order to evaluate the allorejection risk of CAR-engineered cells in more biologically relevant environments. Humanized mouse models, which are generated by engrafting human immune cells or tissues into immune-deficient mice, are often involved in *in vivo* pharmacokinetics and pharmacodynamics (PK/PD) assays.^49^ In this approach, allogeneic therapeutic cells are labeled with tracers like firefly luciferase and enhanced green fluorescence protein (FG) dual reporters for dynamics monitoring. Humanized mice engrafted with human immune cells can be inoculated with therapeutic cells. Throughout the experiment, mice were monitored for survival, and the therapeutic cells were tracked using bioluminescence imaging (BLI).

For instance, in a preclinical study evaluating the host T cell-mediated allorejection of allogeneic CAR-engineered invariant natural killer T (CAR-NKT) cells, humanized NSG mice (NOD scid gamma mice) were injected with human donor-mismatched peripheral blood mononuclear cells (PBMCs) and FG-labeled allogeneic CAR-NKT cells.^10^ BLI was employed to monitor the PK/PD of allogeneic CAR-NKT cells in the presence or absence of donor-mismatched PBMCs. The results demonstrated that allogeneic CAR-NKT cells were able to survive and expand effectively, even in the presence of donor-mismatched PBMCs, suggesting resistance to host T cell-mediated allorejection.^10^ This capacity is likely attributed to their hypoimmunogenic nature, characterized by low surface expression of HLA class I and class II molecules.^11^

In the study evaluating allorejection to KI-A24&E-rejTs, humanized NOG mice (NOD/Shi-scid IL2Rγnull mice) were administered KI-A24&E-rejTs alongside either HLA-A24^+^ NK cells, HLA-A24^−^ NK cells, or no NK cells.^43^ Results demonstrated that KI-A24&E-rejTs persisted effectively in the presence of HLA-A24^+^ NK cells, with no significant difference in bioluminescence signal compared to control groups that received HLA-A24^−^ NK cells and no NK cells.^43^ In addition, FG-labeled KI-A24&E-rejTs were intraperitoneally injected into immunodeficient mice with or without HLA-A24^+^ NK cells and with or without CD8^+^ T cells. BLI revealed that KI-A24&E-rejTs were not eliminated in any group over a 35-day period, indicating that these cells were resistant to allorejection mediated by HLA-A24^+^ NK cells and CD8^+^ T cells.^43^

Overall, both *in vitro* and *in vivo* assays mentioned above serve as non-invasive methods in testing host-cell-mediated allorejection responses. These assays provide a comprehensive evaluation of the CAR-engineered cell products, ensuring their safety and long-term success in preclinical and clinical applications. While promising, the preclinical models used to evaluate allogeneic CAR-T and CAR-NK therapies face several challenges, including the small number of models, xenograft reactivity, and limited relevance for testing persistence. Future efforts should focus on developing advanced models that better simulate human immune environments, particularly in the context of alloimmune responses and long-term engraftment.

On the other hand, to evaluate the presence of pre-existing anti-HLA antibodies and anti-HLA antibody-mediated allorejection responses, complement-dependent cytotoxicity (CDC) assays are routinely employed.^50^ In this assay, donor lymphocytes expressing HLA antigens are incubated with the recipient’s serum in the presence of exogenous complement. If anti-HLA antibodies are present, they bind to the HLA antigens, activating the complement cascade, which induces cell lysis. This cytotoxic effect is quantified through dye exclusion or fluorescence-based techniques, providing a direct measure of anti-HLA antibody levels.^50^ In addition to CDC, other detection methods include ELISA and multiplex bead-based assays, where HLA antigen-coated fluorescent beads capture patient anti-HLA antibodies.

## Strategies to mitigate allorejection

### Ablation of HLA molecules and overexpression of NK inhibitory ligands

**Figure 3:**
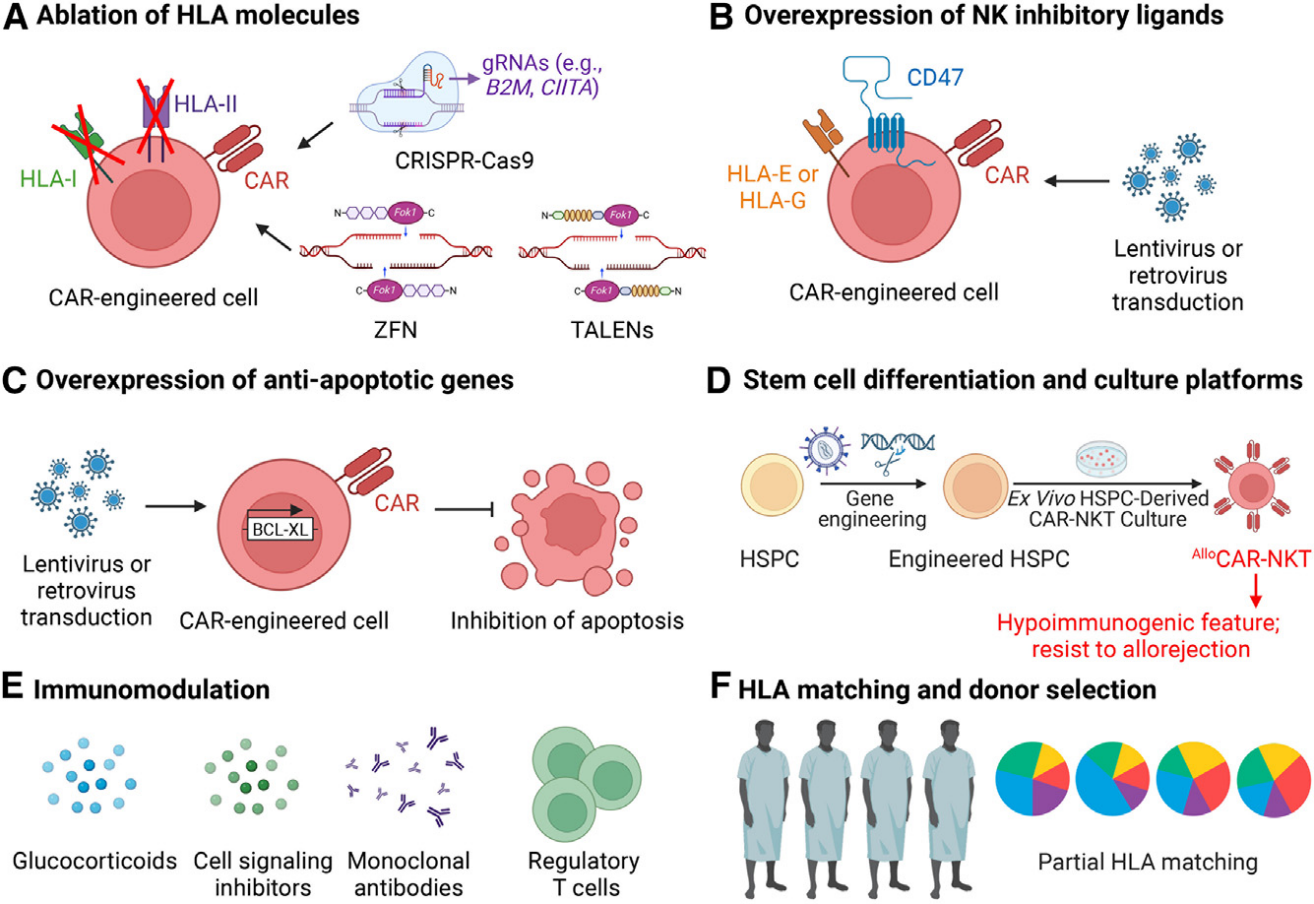
Strategies to mitigate host-cell-mediated allorejection Various strategies have been employed to mitigate host-cell-mediated allorejection of off-the-shelf CAR-engineered therapeutic cells. These include genetic engineering techniques, such as CRISPR-Cas9, zinc-finger nuclease (ZFN), or transcription activator-like effector nucleases (TALENs), to delete HLA-I and HLA-II molecules (A); overexpression of NK cell inhibitory ligands like HLA-E, HLA-G, and CD47 (B); and the incorporation of anti-apoptotic molecules such as BCL-XL (C). Additionally, stem cell engineering and differentiation technologies can generate large quantities of CAR-engineered cells with a hypoimmunogenic phenotype, making them resistant to host-cell-mediated allorejection (D). Immunomodulatory approaches, including the use of glucocorticoids, CSIs, monoclonal antibodies, and regulatory T cells, can also reduce immune rejection (E). Moreover, selecting donors with partial HLA matching can also be an effective strategy in treating allogeneic patients (F).

To mitigate allorejection, various strategies have been developed to address the underlying biological mechanisms, with current approaches primarily involving HLA knockout in therapeutic cells and selecting donors with partially matched HLA profiles (Table 1; Figure 3). When full HLA matching between therapeutic and host cells is not feasible, strategies such as knocking out HLA genes to reduce the recognition of therapeutic cells by the recipient’s immune system or selecting donors with a partial HLA match can be employed to mitigate the risk of immune rejection. The latter strategy exploits a degree of histocompatibility to minimize immune responses without requiring complete HLA matching, a challenge particularly in genetically diverse populations. These approaches collectively aim to diminish immune surveillance and destruction of the therapeutic cells, thereby promoting prolonged cell persistence and improving efficacy in allogeneic settings.

**Table 1:** Strategies to mitigate host-cell-mediated allorejection

Strategy	Advantage	Disadvantage
Ablation of HLAs and overexpression of NK inhibitory ligands	permanent modification precise editing clinical validation	off-target effects
Overexpression of anti-apoptotic genes	preserve patient immune integrity minimize toxicity and infection risks efficient generation of allogeneic cell products crucial for urgent patient needs	risk of oncogenic transformation risk of heightened immune responses
Utilize stem cell culture technology	cell products with high yield and purity versatile genetic modifications	variability based on cell culture process and source clones
Immunomodulation: immunosuppressive agents	extensively used in solid organ transplantation comprehensive guidelines and protocols established	resistance to immunosuppressive agents severe side effects including long-term toxicity, cancer, and metabolic disorders.
Immunomodulation: regulatory T cells	suppress alloreactive T cells protect patient’s immune function long-term tolerance potential	instability in inflammatory environment difficult to obtain sufficient Tregs for treatment
HLA matching and donor selection	high safety profile due to minimum modification reduced manufacturing cost and time	time-consuming donor selection negatively impacting severely ill patients

One key strategy for HLA modification involves genetic engineering approaches, including (1) the deletion of mismatch-causing HLA molecules, and (2) the overexpression of NK cell-inhibiting ligands. With advances in gene-editing technologies, long-term engineering of allogeneic cell sources, including ESCs, HSCs, iPSCs, and allogeneic T cells, has been achieved using tools such as zinc-finger nuclease (ZFN),^51^ transcription activator-like effector nucleases (TALENs),^52^ and the CRISPR-Cas9 system to reduce allorejection risk.^45^^,^^53^ In the case of HLA-I ablation, the most commonly employed technique involves targeting the *B2M* gene,^20^^,^^54^^,^^55^ which is essential for the formation of non-covalent dimers with MHC class I heavy chains. This method effectively eliminates both classical and non-classical HLA-I molecules, thereby significantly reducing the potential for host T cell-mediated rejection.

While the ablation of HLA-A and HLA-B effectively reduces HLA mismatch and T cell-mediated allorejection, the elimination of HLA-C and HLA-E can exacerbate NK cell-mediated rejection. This occurs because both HLA-C and HLA-E engage with inhibitory receptors on NK cells, protecting target cells from NK cell-mediated lysis.^56^ HLA-C interacts with KIR receptors, delivering inhibitory signals that prevent NK cell cytotoxicity; its knockout would lead to increased NK cell activity. Similarly, HLA-E, a non-polymorphic molecule, engages with both KIR and CD94/NKG2A receptors to inhibit NK cell-mediated lysis. To address the NK-mediate lysis, a strategy combining B2M knockout with HLA-E overexpression has been shown to reduce cell lysis both *in vitro* and *in vivo*.^51^^,^^57^ Additionally, the use of precise CRISPR-Cas9 techniques to selectively target HLA-A and HLA-B while preserving HLA-C expression has successfully generated HLA pseudo-homozygous iPSCs, which are resistant to both T cell- and NK cell-mediated allorejection.^58^

For HLA-II elimination, *HLA-DR* and *CIITA* are the primary targets for knockout. HLA-DR is the key driver of T cell activation in various immune responses. Its ablation using TALENs has been shown to be effective and safe, demonstrating potential for modifying effector cells to prevent host-versus-graft reactions in cancer immunotherapy, even under IFN-γ stimulation.^52^ CIITA, a transcriptional coactivator essential for the expression of all MHC-II molecules (HLA-DR, HLA-DP, HLA-DQ), provides a broader approach. Disrupting CIITA results in a significant reduction in surface expression of all MHC-II molecules, impairing CD4^+^ T cell activation and reducing alloreactivity in both *in vitro* and *in vivo* studies.^59^^,^^60^

In addition to these strategies, recent studies have shown that overexpressing CD64 provides a protective advantage to CAR-T cells against both HLA and non-HLA antibody-mediated cytotoxicity.^61^ CD64 is a high-affinity receptor for the Fc region of immunoglobulin G (IgG), which allows CAR-T cells to effectively capture IgG Fc. By binding to IgG Fc, CD64 sequesters donor-specific antibodies (DSAs), making them unavailable to effector cells and complement components. This mechanism enables CAR-T cells to evade ADCC and CDC from host effector cells.^61^ As a result, this approach adds an additional layer of defense for CAR-T cells against rejection by the host immune system.

Gene editing is a critical tool in the development of off-the-shelf cell therapies, achieved via viral and non-viral delivery platforms, including systems like CRISPR-Cas9, ZNF, and TALENs. These methods have been employed to delete HLA molecules in iPSC-derived cells, such as T cells and macrophages.^62^^,^^63^ Viral platforms, such as lentivirus, retrovirus, and adeno-associated virus (AAV), offer high delivery and editing efficiency, particularly in hard-to-transfect cells like primary and stem cells. However, they carry risks, including insertional mutagenesis, especially with integrating viruses (e.g., lentivirus) and are limited in genetic payload capacity. Non-viral platforms, on the other hand, utilize physical or chemical methods to introduce gene-editing components. Key examples include lipid nanoparticles (LNPs) and polymer-based nanoparticles.^64^^,^^65^ LNPs encapsulate RNA or DNA, such as mRNA encoding CRISPR-Cas9, offering a non-integrating, safer approach commonly used for *in vivo* delivery. Polymer-based nanoparticles, such as those formed with polyethyleneimine, facilitate DNA or RNA uptake with reduced toxicity and controlled release. While non-viral methods lower risks of immune responses and insertional mutagenesis, they generally exhibit lower efficiency than viral vectors and often require optimization for different cell types or conditions. In summary, viral platforms provide higher efficiency but present risks of genome integration and immune reactions, whereas non-viral approaches offer safer, transient editing with the trade-off of requiring more complex optimization for effective delivery.^64^^,^^65^ The choice of platform depends on the specific application, target cell type, and safety requirements.

Gene-editing tools such as CRISPR-Cas9 and TALENs have been utilized in clinical trials to knock out HLA genes in allogeneic therapeutic cells (Table 2). TALENs are recognized for their high specificity, precision, and reduced off-target mutation rates, making them particularly suitable for applications where safety and accuracy are critical.^83^ In contrast, CRISPR-Cas9 offers advantages in terms of efficiency, ease of design, scalability, and the capacity to perform multiplexed gene targeting, making it more appropriate for complex or large-scale gene-editing endeavors.^83^ As CRISPR technology advances, its versatility and adaptability render it increasingly favorable in clinical applications, while TALENs remain valuable in contexts that demand maximal precision and minimal off-target effects.

**Table 2:** Reported clinical trials of allogeneic CAR-T cell therapy

Cell product (year/reference)	Patient and disease	CAR target	Other gene-engineering strategy	Therapy design	Clinical outcome	Related preclinical or clinical study (year/reference)
UCART19, allogeneic CD19-targeting CAR-T (2020^66^)	7 pediatric and 14 adult patients with B-ALL	CD19	lentiviral transduction of genes that encode RQR8 as a safety switch; TCRαβ and CD52 were KO by TALENs	CAR-T cells were infused at escalating doses (6 × 10^6^, 6-8 × 10^7^, or 1.8–2.4 × 10^8^ cells/kg of body weight) and into children at 1.1–2.3 × 10⁶ cells/kg of body weight post lymphodepletion with fludarabine and cyclophosphamide, with or without alemtuzumab	grade 1–2 CRS was observed in 91% of patients; grade 3–4 CRS was observed in 14%; neurotoxicity was observed in 38%; grade 1 skin GvHD was observed in 10%; prolonged cytopenia was observed in 32%; two treatment-related deaths occurred due to neutropenic sepsis and pulmonary hemorrhage; patients without alemtuzumab showed no CAR-T expansion or anti-leukemic activity; infused CAR-T cells expanded and persisted for a median duration of 4.1 months in 71% of patients; CR was achieved in 67% patients	UCART19 cells persisted until conditioning prior to a successful allogeneic stem cell transplantation, and Molecular remissions were achieved within 28 days in two infant patients (2017^67^)
ALLO-501, allogeneic CD19-targeting CAR-T (2021^68^)	47 patients with relapsed or refractory non-Hodgkin’s lymphoma (46 patients received allogeneic cell therapy infusion)	CD19	disruption of TRAC and CD52 gene using TALENs	CAR-T cells were infused at escalating doses (4 × 10^7^ cells, 1.2 × 10^8^ cells, or 3.6 × 10^8^ cells/kg of body weight) post lymphodepletion with anti-CD52 monoclonal antibody (mAb), fludarabine, and cyclophosphamide	no dose-limiting toxicities or GvHD were observed; no immune effector cell-associated neurotoxicity syndrome (ICANS) were observed; grade 1–2 CRS was observed in 21.7% of patients; cytopenia was observed in 82.6%; grade 3+ infections were observed in 23.9%; 5 patients died after treatment; ORR was 75%, and CR was achieved in 50%; the longest observed ongoing CR lasted for over 15 months	as shown above (2017^67^)
ALLO-501A, allogeneic CD19-targeting CAR-T (2021^69^)	20 patients with relapsed or refractory large B cell lymphoma (15 patients received allogeneic cell therapy infusion)	CD19	disruption of TRAC and CD52 gene using TALENs	CAR-T cells were infused at escalating doses (4 × 10^7^ or 1.2 × 10^8^ cells/kg of body weight) post lymphodepletion with anti-CD52 mAb, fludarabine, and cyclophosphamide	no CRS, GvHD, ICANS, dose-limiting toxicities, or grade 3+ infections were observed; cytopenia was observed in 72% of patients; ORR was 50%, and CR was achieved in 50%; for patients with consolidation, both ORR and CR rate were 66.7%; therapeutic cells expanded after the second round of infusion	as shown above (2017^67^)
Allogeneic CD7-targeting CAR-T from HLA-matched or haploidentical donors (2021^70^)	20 patients with relapsed or refractory T cell acute lymphoblastic leukemia	CD7	N/A	CAR-T cells were infused at the dose of 0.5 or 1 × 10^6^ CAR-T cells/kg post lymphodepletion with fludarabine and cyclophosphamide	CRS grades 1–2 occurred in 90% of patients and grades 3–4 in 10%; grade 3–4 cytopenia was observed in 100% of patients; neurotoxicity grades 1–2 occurred in 15%; GvHD grades 1–2 in 60%; and viral activation grades 1–2 in 20%; complete remission was achieved in 90% of patients; CAR-T cells remained detectable in all five patients assessed at 6 months post infusion	N/A
UCART19, allogeneic CD19-targeting CAR-T (2022^71^)	25 patients with relapsed or refractory B-ALL	CD19	lentiviral transduction of genes that encode RQR8 as a safety switch; TCRαβ and CD52 were KO by TALENs	CAR-T cells were infused into at one of escalated doses (6 × 10^6^ cells, 6–8 × 10^7^ cells, or 1.8–2.4 × 10^8^ CAR-T cells per kilogram of body weight) post lymphodepletion with fludarabine and cyclophosphamide with or without alemtuzumab	dose-limiting toxicities were observed in 12% of patients; grade 3+ CRS was observed in 24%; prolonged cytopenia was observed in 8%; grade 3+ neurotoxicity was observed in 4%; grade 3+ infections were observed in 28%; grade 1 acute skin GvHD was observed in 8%; 16% of patients died due to CAR-T or lymphodepletion; ORR was 48%; median relapse-free survival was 7.4 months; median progression-free survival was 2.1 months; median overall survival was 13.4 months	as shown above (2017^67^)
CTX110, allogeneic CD19-targeting CAR-T (2022^72^)	34 patients with relapsed or refractory large B cell lymphoma (32 patients received allogeneic cell therapy infusion)	CD19	TRAC and B2M gene were disrupted by CRISPR-Cas9	CAR-T cells were infused at a dose range of 3–6 × 10^8^ cells per patient post lymphodepletion with fludarabine and cyclophosphamide	best ORR and CR were 67% and 41%; 6-month CR was achieved in 19% of patient; CAR-T expansion and deepened clinical response were observed in patients with the second infusion; no GvHD was observed; grade 1–2 CRS was observed in 56% of patients; ICANS was observed in 9.4%; nearly half of all patients who achieved a CR maintained it for at least 6 months	N/A
CTA101, allogeneic CD19/CD20 dual-targeting CAR-T (2022^24^)	6 patients with relapsed or refractory acute lymphoblastic leukemia	CD19 and CD22	TRAC and CD52 were disrupted; suicide switch RQR8 were added	CAR-T cells were infused at one of two doses (1 × 10^8^ or 3 × 10^8^ cells/kg of body weight) post lymphodepletion with cyclophosphamide, fludarabine, and alemtuzumab	grade 1–2 CRS was observed in 83.3% of patients; grade 3 CRS was observed in 16.7%; grade 3+ infections were observed in 50%; cytopenia was observed in 50%; CR was observed in 83.3%; therapeutic cell expansion was observed in CR patients post day 28 infusion	CAR-T cells efficiently targeted tumor cells, did not mediate graft-versus-host reactions and were rendered resistant to destruction by alemtuzumab (2015^73^)
P-BCMA-ALL01, allogeneic BCMA-targeting CAR-T (2023^74^)	24 patients with relapsed or refractory multiple myeloma	BCMA	iCas9 safety switch was added by non-viral transposon-based integration	CAR-T cells were infused at one of seven escalated dose levels (from 6.25 × 10^4^ to 1.5 × 10^7^ cells/kg of body weight) post lymphodepletion with fludarabine and cyclophosphamide, with or without alemtuzumab	no dose-limiting toxicities or GvHD were observed; grade 3+ treatment-emergent adverse events were observed, including neutropenia (36%), leukopenia (32%), and anemia (23%); grade 1 CRS was observed in 14% of patients; grade 1 ICANS was observed in 4%	N/A
WU-CART-007 1001, allogeneic CD7-targeting CAR-T (2023^75^)	9 patients with relapsed or refractory T cell acute lymphoblastic leukemia and 3 patients with lymphoma	CD7	TRAC was deleted	CAR-T cells were infused at one of four doses (1 × 10^8^, 3 × 10^8^, 6 × 10^8^, or 9 × 10^8^ cells per patient) post lymphodepletion with fludarabine and cyclophosphamide	CRS was observed in 67% of patients; grade 1 ICANS was observed in 8.3%; no GvHD or prolonged T cell aplasia was reported; no pancytopenia was reported; ORR at ≥ dose level (DL) 2 of evaluable patients was 43%; duration of response extending to 86 days was reported	CAR-T cells showed strong cytotoxicity against CD7-expressing cells, and no cytotoxicity \ against hematopoietic progenitor cells (2021^76^)
Allogeneic CD7-targeting CAR-T from HLA-matched or haploidentical donors (2023^77^)	10 patients with relapsed or refractory T cell malignancies (5 patients received allogeneic cell therapy infusion)	CD7	N/A	CAR-T cells were infused at the dose of 1 or 2 × 10^6^ CAR-T cells/kg post lymphodepletion with fludarabine and cyclophosphamide; 5 patients received allogeneic CAR-T and 5 received autologous CAR-T	no dose-limiting toxicity or neurotoxicity was observed; patients treated with allogeneic CAR-T cells had higher remission rate, lower recurrence and more sustained CAR-T cell persistence than those receiving autologous products	donor-derived CD7-targeting CAR-T cells exhibited efficient expansion and achieved a high CR rate with manageable safety profile (2021^70^)
ALLO-715, allogeneic BCMA-targeting CAR-T (2023^78^)	43 patients with relapsed/refractory multiple myeloma (MM)	BCMA	TRAC and CD52 KO using TALENs	CAR-T cells were infused at one of four doses (40 × 10^6^, 160 × 10^6^, 320 × 10^6^, 480 × 10^6^) into patients post lymphodepletion with fludarabine and cyclophosphamide combined with anti-CD52 antibody	grade ≥3 adverse events were observed in 88.0% of patients; CRS occurred in 55.8% of patients, with 2.3% experiencing grade ≥3. neurotoxicity was reported in 14% of patients, with no grade ≥3; infections were documented in 53.5% of patients, with 23.3% presenting grade ≥3; in patients receiving 320 × 10^6^ cells, 70.8% demonstrated a clinical response, with 45.8% achieving a very good partial response or better, including 25% who attained a complete or stringent complete response	as shown above (2017^67^)
UCART22, allogeneic CD22-targeting CAR-T (2023^79^)	21 patients with relapsed or refractory B-ALL	CD22	TRAC and CD52 were KO by TALENs	CAR-T cells were infused at an escalated dose starting from 1 × 10^6^ cells/kg of body weight post lymphodepletion with alemtuzumab, fludarabine, and cyclophosphamide	no grade 3+ CRS, dose-limiting toxicity, or ICANS were observed; the response rate at DL2 with CAR-T cells was 67%, compared to 50% at DL3	as shown above (2017^67^)
TyU19, allogeneic CD19-targeting CAR-T (2024^80^)	1 patient with refractory immune-mediated necrotizing myopathy and 2 patients with systemic sclerosis	CD19	HLA-A, HLA-B, CIITA, TRAC, and PD-1 were KO by CRISPR-Cas9	CAR-T cells were infused at 1 × 10^6^ cells/kg of body weight post lymphodepletion with fludarabine and cyclophosphamide	no CRS was observed; no GvHD-related symptoms were observed; Ig levels were maintained above normal levels	N/A
Allogeneic CD19/CD22 dual-targeting CAR-T (2024^81^)	2 patients with B-ALL	CD19 and CD22	N/A	CAR-T cells were infused at 3 × 10⁶ cells/kg of body weight post lymphodepletion with fludarabine and cyclophosphamide, followed by a re-infusion of 6 × 10^6^ cryopreserved cells/kg of body weight if relapsed	N/A	N/A
CTX130, allogeneic CD70-targeting CAR-T (2024^82^)	16 patients with advanced clear cell renal cell carcinoma	CD70	insert the CAR into the TRAC locus; disrupt β2-microglobulin; disrupt CD70	CAR-T cells were infused at one of four doses (30 × 10^6^, 100 × 10^6^, 300 × 10^6^, 900 × 10^6^) into patients post lymphodepletion with fludarabine and cyclophosphamide	no patients experienced dose-limiting toxicity, and disease control was achieved in 81.3% of cancer patients	CTX130 and its modified version with Regnase-1 and TGFβR2 knockout had potent expansion and antitumor efficacy (2024^82^)

Studies are listed chronologically. KO, knocked out; CR, complete remission; N/A, not available.

Despite promising results in preclinical and clinical investigations, concerns persist regarding the use of genetic editing tools to enable therapeutic cells to evade the host’s immune surveillance. The most significant issue is the potential off-target effects that may introduce mutations or disrupt critical genes, potentially leading to disease or other adverse outcomes.^84^ Understanding these dynamics is essential for ensuring the safety and efficacy of genetic editing approaches in clinical applications.

### HLA matching and donor selection

HLA matching remains a cornerstone in the selection of donors for transplantation and immunotherapy, as it reduces the incidence of GvHD by aligning the recipient’s immune system with the donor’s tissue antigens. Full HLA compatibility between donor and recipient is considered ideal, yet achieving this across diverse populations presents a significant challenge due to the extensive polymorphism in HLA loci, particularly in the key regions HLA-A, HLA-B, HLA-C, and HLA-DRB1.^85^ In clinical practice, partial HLA matching, where only a subset of HLA alleles is shared, has emerged as a viable strategy to expand the donor pool, particularly in urgent settings or for large-scale therapeutic applications. Although suboptimal, partial matches of cell therapeutic products from CAR-T cells to mesenchymal stem cells have demonstrated efficacy in both clinical trials and preclinical models without triggering severe allorejection response.^77^ Furthermore, the integration of advanced gene-editing technologies, such as CRISPR, and the use of iPSCs have further enhanced the feasibility of generating immune-compatible cells that can tolerate partial HLA mismatches.^63^^,^^86^

### Overexpression of anti-apoptotic genes

Apoptotic cell death, regulated by the BCL-2 family, has been identified as a major factor contributing to the loss of transplanted stem cells. This family encompasses both pro-apoptotic proteins, including BAX, BAK, BIM, PUMA, and BMF, as well as anti-apoptotic members such as BCL-2, BCL-XL, MCL-1, BFL-1, and BCL-W.^87^ Among these, BCL-XL has garnered attention for its dual role in modulating both mitochondrial-mediated intrinsic apoptosis and receptor-triggered extrinsic cell death pathways, making it a promising candidate for reducing graft failure.^87^ Preclinical studies have demonstrated that overexpression of BCL-XL improves cell survival in several models, including hematopoietic stem cell transplantation (HSCT) and bone marrow transplantation.^88^

Additionally, survivin, a member of the inhibitor of apoptosis protein (IAP) family, plays a crucial role in apoptosis inhibition and cell cycle regulation.^89^ Animal models have shown that survivin enhances engraftment and prolongs allograft survival, notably in islet cell transplants, fully mismatched renal transplants, and HSCT.^90^^,^^91^

Despite encouraging preclinical evidence, the clinical application of anti-apoptotic gene therapies in allogeneic cell transplantation remains limited. Concerns persist regarding the potential link between overexpression of anti-apoptotic genes and oncogenesis, as well as the risk of heightened immune responses, particularly through T and B lymphocyte activation.^92^ Therefore, further investigation is required to validate the safety and efficacy of this approach in the clinical setting and to optimize its integration into allogeneic cell therapeutic protocols.

### Stem cell differentiation and culture platforms

In addition to genetic engineering, stem cell platforms such as hematopoietic stem and progenitor cells (HSPCs) and iPSCs have been employed to generate low-immunogenicity cells for allogeneic CAR-T therapy. These platforms generate cells exhibiting intrinsically lower MHC expression, likely due to specialized culturing conditions. For HSPC platforms, Li et al. developed allogeneic NKT cells using a 3D artificial thymic organoid (ATO) model, which displayed reduced HLA class I and II expression and lower levels of NK-activating ligands, such as ULBP-1.^12^ These cells were resistant to T and NK cell-mediated rejection, as demonstrated by *in vitro* mixed lymphocyte assays and *in vivo* pharmacokinetic studies, while maintaining potent cytotoxic activity.^12^

Subsequently, the same group developed allogeneic interleukin (IL)-15-enhanced CAR-engineered NKT (^Allo15^CAR-NKT) cells from HSPCs using a feeder-free, serum-free culture system, which retained a similar hypoimmunogenic profile.^11^ RNA sequencing (RNA-seq) analysis confirmed stable hypoimmunogenicity, linked to downregulated expression of genes involved in HLA and NK ligand.^11^ Methylation sequencing further revealed hypermethylation in the promoter regions of HLA and NK ligand genes, alongside a desensitized IFN-γ-JAK-STAT1 signaling pathway, marked by low STAT1 phosphorylation under both basal and IFN-γ-stimulated conditions.^11^

For the iPSC platform, Wang et al. employed iPSCs reprogrammed from CD62L^+^ naive and memory T cells to generate allogeneic CD19 CAR-T cells with reduced HLA expression.^93^ These iPSC-derived T cells also exhibited lower MHC gene expression compared to conventional CAR-T cells, without showing signs of exhaustion. Gene set enrichment analysis revealed downregulation of MYC target genes and IFN-γ response signatures in iPSC-derived CD19 CAR-T cells compared to conventional CD19 CAR-T cells.^93^ The lower MHC expression and skewed CD8^+^ cell population observed in iPSC-derived CAR-T cells may result from initiating with naive and memory T (Tn/mem)-derived iPSC clones or the lack of thymic epithelial cell influence during culture.^93^

While stem cell platforms offer a promising approach to generating low-immunogenicity cells without genetic engineering, the outcomes are largely influenced by the culture conditions and cell source. Despite progress, fully eliminating HLA mismatches remains difficult. Incorporating genetic engineering techniques could provide a more precise solution for HLA elimination, enhancing compatibility and reducing the risk of immune rejection in allogeneic CAR therapies. It is important to note that, while these stem cell-based methods hold potential, they are still in the preclinical stages, and no clinical trials involving allogeneic cell products derived from these platforms have been reported. Continued innovation and rigorous evaluation will be crucial in bridging the gap from preclinical promise to clinical application.

### Immunomodulation

Immunomodulation strategies have been extensively employed in allogeneic therapies, particularly in allogeneic HSCT (allo-HSCT), to mitigate GvHD.^94^ These approaches include the use of immunosuppressive drugs, such as tacrolimus, cyclosporine, and mycophenolate mofetil, to dampen immune responses, as well as T cell depletion from the graft to lower the risk of GvHD.^94^ Additionally, post-transplant cyclophosphamide has been utilized to selectively reduce GvHD while preserving the beneficial graft-versus-leukemia effects. The adoptive transfer of regulatory T cells (Tregs) has also shown promise in modulating immune responses and preventing GvHD.^94^ These immunomodulation techniques in allo-therapy have provided valuable insights for addressing allorejection by attenuating host immune responses to allogeneic therapeutic cells. The reduction of allorejection can be achieved through pharmacological or biological methods. Pharmacologically, immunosuppressive regimens often include glucocorticoids, cell signaling inhibitors, and monoclonal antibodies.^95^ Despite their varied mechanisms of action, these agents predominantly target T and B lymphocytes, central mediators of immune rejection. In contrast, biological strategies exploit immune-regulatory cells, such as Tregs, to suppress effector T cells and mitigate alloreactive immune responses.^96^

Glucocorticoids, key mediators of the stress response, exhibit potent anti-inflammatory and immunosuppressive effects through the regulation of gene expression in leukocytes.^97^ Upon binding to glucocorticoid receptors (GRs), the receptor-ligand complex translocates into the nucleus, modulating the transcription of genes involved in inflammatory pathways. Glucocorticoids downregulate pro-inflammatory mediators, including IL-2, tumor necrosis factor (TNF)-α, chemokines, and cell adhesion molecules, while upregulating inhibitory cytokines such as IL-10 and IL-1 receptor antagonist.^97^ Additionally, GR activation inhibits transcription factors like nuclear factor κB (NF-κB) and AP-1, further suppressing immune activation, particularly in the context of allogeneic stem cell transplantation. Clinical trials have demonstrated that low-dose glucocorticoids can significantly reduce the incidence of GvHD following allogeneic hematopoietic cell transplantation.^98^ Despite their efficacy, prolonged glucocorticoid treatment is associated with adverse metabolic effects, including osteoporosis, hypertension, dyslipidemia, and insulin resistance. Moreover, a subset of acute GvHD patients is steroid resistant, leading to poor clinical outcomes in these individuals.^99^

Immune responses are carried out through various complex and interrelated signaling pathway, therefore by interrupting these pathways through cell signaling inhibitors (CSIs), allorejection can be mitigated. Various CSI, including Janus kinase (JAK) inhibitors, calcineurin inhibitors, mechanistic target of rapamycin (mTOR) inhibitors, inosine 5′-monophosphate dehydrogenase (IMPDH) inhibitors, TNF-α-converting enzyme (TACE) inhibitors, rho-associated protein kinase (ROCK) inhibitors, or IL-1 receptor-activated kinase 4 (IRAK4) have been developed to modify the immune pathways.^95^^,^^100^ Among these, the two most prominent ones are calcineurin inhibitors and mTOR inhibitors.

Calcineurin is a protein phosphatase involved in the NFAT signaling pathway, specifically the activated calcineurin dephosphorylates the NFAT transcription factors, enabling it to localize in the nucleus. Calcineurin inhibitors (CNIs) interrupt this process by binding to a type of cytosolic proteins called immunophilins. This complex consequently binds to calcineurin, preventing from becoming activated by interacting with Ca^2+^/calmodulin. Consequently, there is a lack of activated calcineurin to dephosphorylate NFA to translocate into the nucleus, hence inhibiting the NFAT-dependent gene expression. This includes many important cytokines such as IL-2, IFN-γ, and TNF-α, as well as cell surface molecules such as CD40L, CD95, and CD25.^101^ The overarching result from these affects is reduced T cell proliferation and proinflammation cytokine production. Beyond the NFAT pathway, CNIs also attenuate downstream TCR signaling by inhibiting the phosphorylation of pivotal proteins such as ZAP70 and LAT, further curbing T cell activation.^101^ Clinically, CNI is the standard treatment for solid organ transplant and allogeneic HSCT, but it comes with severe side effects, inducing neurological complications, nephrotoxicity, hypertension, dyslipidemia, and new onset of diabetes.^102^

The mTOR is a conserved serine/threonine protein kinase essential for regulating cellular metabolism, growth, proliferation, and survival. mTOR operates within two distinct complexes: mTORC1, which predominantly controls cell growth and metabolism, and mTORC2, which governs cell proliferation and survival.^103^ Inhibitors of mTOR (mTORis), such as rapamycin, specifically target mTORC1, resulting in reduced CD8^+^ T cell proliferation and Th1 cytokine production. This mechanism promotes an immune environment more tolerant to allografts by increasing Tregs while decreasing alloreactive T cells.^104^ Clinically, mTOR inhibitors have been extensively used to prevent GvHD in solid organ transplantation and are increasingly applied in HSCT, as demonstrated by positive clinical outcomes reducing GvHD incidence.^105^

Monoclonal antibodies that can bind to various receptors and cytokines are also being utilized to inhibit T cell and B cell activation. Three prominent antibodies are basiliximab, adalimumab, and rituximab. Specifically, basiliximab is a chimeric monoclonal antibody that binds to the alpha-unit of IL-2 receptors on the surface of activated T lymphocytes to reduce proliferation,^106^ adalimumab binds TNF-α to prevent TNF-α receptors and thus reduce immune activation in solid organ transplant patients,^107^ and rituximab binds to the CD20 expressed on B cells to induce the killing B cells.^108^ Although the use of monoclonal antibodies to reduce rejection following allogeneic cell treatment is not yet prevalent, its successes in treating solid organ transplant and other immune diseases have shown promising potential.

In conclusion, glucocorticoids and various CSIs are integral to the modulation of immune responses, particularly within the contexts of transplantation and allogeneic stem cell therapies. These immunosuppressive agents are frequently employed concurrently as a first-line strategy to mitigate the risk of rejection in both cell and solid organ transplantations. While established guidelines and protocols underpin their use, the effectiveness of these agents can vary across patient populations, and they are often associated with significant complications due to their systemic impact on the host immune system. Consequently, there is an urgent need for tailored therapeutic approaches that not only minimize adverse effects but also optimize efficacy in preventing allorejection, thereby enhancing transplant outcomes and improving patient quality of life.

In addition to immunosuppressive regimens, investigating the role of Tregs in mitigating allorejection is of significant importance. Tregs are a subset of human immune cells that specialize in restraining excessive immune responses and inducing peripheral immune tolerance. This function makes them a promising candidate to address allorejection occurring from allogeneic cell treatment. Specifically, Tregs that express CD4^+^CD25^+^CD127^−^FOXP3^+^ are the main population used to develop adoptive Treg transfer and, endogenously, they originate either from thyroid or peripheral naive CD4^+^ cells after encountering antigen and contact with cytokines, such as TGF-β and IL-2.^109^ Both subsets can suppress inflammatory alloreactive T cells through both contact- and non-contact-dependent mechanisms, which are categorized as cytolysis, secretion of inhibitory cytokines, metabolic disruption, and targeting of dendritic cells (DCs).^96^ Tregs are efficient at suppressing alloreactive T cells but they only account for 5%–10% of CD4^+^ T cells in peripheral blood. Therefore, the quantity of Tregs directly isolated from donors is significantly insufficient to meet the demands for clinical infusion, and the quality of cells are also suboptimal. Therefore, various strategies to expand autologous Tregs have been developed, including *ex vivo* expansion using αCD3/αCD28 beads and IL-2 stimulation and *in vivo* expansion with IL-2 only.^96^ Furthermore, allogeneic Tregs derived from umbilical cord blood or pediatric thymuses also exhibit ability to inhibit GvHD development, but with suboptimal effects.^110^ Clinically, various studies have examined the efficacy of adoptive transfer of Tregs after allo-HSCT and have reported reduced incidence of GvHD with no increase in risks of infection, relapse, or early mortality.^111^ Despite promising clinical results, there are several major concerns regarding Tregs, specifically their instability in inflammatory environments, where they are prone to losing FOXP3 expression and thus losing their immunosuppressive ability.^112^ In addition, obtaining enough cells to transfer remains challenging; thus, universal Treg products are urgently needed but require additional manufacturing, such as knocking out classical HLA molecules and knocking in non-canonical HLA molecules.^113^

Of note, given the robust ability of Tregs to suppress alloreactive T cells, concerns arise about whether they might also inhibit the function of therapeutic cells, thereby reducing their tumor-killing efficacy. However, several studies have demonstrated that CD8^+^ T cells activated for leukemia treatment are not significantly impacted by the presence of Tregs.^114^^,^^115^ To mitigate any residual concerns, strategies that genetically engineer allogeneic therapeutic cells to resist Treg-mediated suppression could be explored. One promising approach is the use of CARs deficient in Lck signaling, which have been shown to resist Treg suppression.^116^ Additionally, modifications such as expressing inhibitory molecules like CTLA-4 or PD-1 or knocking out HLA molecules could be investigated to enhance the ability of therapeutic cells to evade Treg suppression. Continued research into these approaches is necessary to determine whether they can successfully preserve the efficacy of therapeutic cells while mitigating host-cell-mediated allorejection, ultimately improving outcomes in allogeneic therapies.

## Clinical trial experience using allogenic cell products

### Therapeutic cell types

Currently, three allogeneic therapeutic cell types, namely T cells, NK cells, and NKT cells, have been studied in clinical trials, demonstrating promising therapeutic efficacy and safety (Table 2). The majority of allogeneic cell therapies involve conventional CAR-T cells, building on extensive experience from autologous CAR-T cell therapy.^4^^,^^117^^,^^118^ Allogeneic CAR-T cells have been extensively evaluated in preclinical and clinical studies, with the primary safety concern being the risk of GvHD.^19^^,^^119^^,^^120^ To address this, advanced gene-editing strategies, such as knocking out *TRAC* and *TRBC* genes and knocking in CAR constructs into the *TRAC* locus, have been applied.^21^^,^^23^^,^^84^^,^^121^ However, endogenous TCRs provide certain benefits for CAR-T cell function and persistence *in vivo*, suggesting that preserving intact TCRs may be advantageous.^122^ Therefore, future gene-engineering strategies should focus on mitigating GvHD risk while maintaining TCR-mediated functionality.

Allogeneic CAR-NK cells have also been explored in clinical trials, such as a trial using cord blood-derived CAR-NK cells that were partially HLA-matched with the recipient to treat CD19-positive non-Hodgkin’s lymphoma or chronic lymphocytic leukemia.^123^ CAR-NK cells naturally induce lower GvHD risk and fewer adverse effects, such as cytokine release syndrome (CRS) and neurotoxicity.^124^^,^^125^^,^^126^ However, CAR-NK cells face challenges related to limited *in vivo* persistence and the inability to be cryopreserved for long-term storage.^124^^,^^125^^,^^126^ In this phase I clinical trial, CAR-NK cells were freshly infused into cancer patients 15 days post culture, which adds complexity to the manufacturing process and requires close coordination between manufacturing and clinical operations.^123^ To enhance *in vivo* persistence, immune-stimulatory genes such as IL-15 have been included in CAR-NK cell products, resulting in improved performance *in vivo*.^123^

An allogeneic CD19-targeting CAR-NKT cell product has also been investigated in a recent clinical trial for the treatment of relapsed or refractory B cell malignancies.^25^ NKT cells exhibit potent antitumor activity and can target multiple tumor mechanisms.^29^^,^^127^^,^^128^ Notably, these cells recognize the non-polymorphic MHC-like molecule CD1d, which eliminates the risk of GvHD, making them suitable for off-the-shelf cancer therapies.^129^^,^^130^^,^^131^ CAR-NKT cells were engineered with IL-15 to enhance their *in vivo* performance, showing improved outcomes in both preclinical and clinical studies.^25^^,^^132^^,^^133^ However, IL-15 has also been associated with increased CRS in conventional CAR-T cell therapies, as demonstrated in a trial targeting glypican-3 (GPC3) in hepatocellular carcinoma (HCC).^134^ Beyond IL-15, other cytokines, such as IL-12, IL-18, and IL-21, have been evaluated in preclinical studies, showing potential to enhance CAR-engineered cell therapies.^4^ Moving forward, selecting the most effective and safest immune-enhancing strategies for CAR-engineered cell therapies will be critical in optimizing their clinical applications and therapeutic efficacy.

Other cell types are emerging as promising avenues for advancing allogeneic cell therapies, including gamma delta (γδ) T cells, virus-specific allogeneic T cells, and iPSC-derived NK/T cells showing particular potential. Like NKT cells, γδ T cells do not depend on MHC-mediated antigen presentation, enabling the bypass of HLA matching, which is an important advantage for allogeneic applications. A current clinical trial is investigating the use of allogeneic Vγ9Vδ2 T cells to treat acute myeloid leukemia (AML), specifically assessing the safety and feasibility of infusing γδ T cells from haploidentical donors into patients with refractory or relapsed AML after lymphodepletion.^135^ The trial reported no serious treatment-related adverse events or grade 3 or higher toxicities, confirming the safety of γδ T cell infusions at doses up to 10^8^ cells per kg body weight per patient.^135^ Nonetheless, the small sample size and the role of lymphodepleting chemotherapy in the observed responses remain important areas for further investigation.

Virus-specific T cells (VSTs) derived from healthy donor blood have emerged as a therapeutic option for treating virus-associated diseases and malignancies, particularly in immunocompromised individuals such as those who have undergone HSCT or organ transplantation.^136^ Clinical applications of donor-derived VSTs have shown promising outcomes in managing post-transplant lymphoproliferative disorder^137^ and refractory viral infection post HCT,^138^ demonstrating a favorable safety profile with limited toxicities. Despite these successes in viral infections post transplant, the exploration of allogeneic VSTs in the treatment of virus-driven cancers remains limited, indicating a need for further clinical trials to assess their efficacy in oncological settings.

iPSCs provide a promising platform for allogeneic cell therapies due to their capacity for multiplex gene editing.^139^ iPSCs can be engineered to knock out HLA molecules or overexpress NK inhibitory ligands, enhancing immune evasion.^58^^,^^140^ They can also be modified to express molecules like CD16 for improved ADCC or cytokines such as IL-2 and IL-15 for enhanced *in vivo* expansion and persistence. Furthermore, iPSCs eliminate donor variability and offer an unlimited supply, making them ideal for large-scale production of allogeneic NK and T cells for off-the-shelf therapies. iPSC-derived CAR-T cells have demonstrated tumor control in preclinical studies,^141^ and clinical trials investigating iPSC-derived TCR-knockout CAR-T cells is currently ongoing, highlighting the potential of this approach in cancer treatment.^142^

### Clinical strategies to manage allorejection

Current allogeneic CAR-engineered cell products utilize a range of gene-engineering strategies to mitigate host-cell-mediated allorejection, including lymphodepletion regimens, partial HLA matching, and the knockout of HLA-I and HLA-II molecules (Tables 2 and 3). These approaches aim to improve the persistence and efficacy of allogeneic cell therapies by reducing the risk of immune rejection.

**Table 3:** Reported clinical trials of allogeneic CAR-NK and CAR-NKT cell therapy

Cell product (year/reference)	Patient and disease	CAR target	Other gene-engineering strategy	Therapy design	Clinical outcome	Related preclinical study (year/reference)
HLA-mismatched CD19-targeting CAR-NK (2020^123^)	6 patients with relapsed or refractory CD19-positive non-Hodgkin’s lymphoma, and 5 patients with chronic lymphocytic leukemia	CD19	cord blood-derived NK cells were transduced with a retroviral vector expressing genes that encode CAR, IL-15, and inducible caspase 9 as a safety switch	CAR-NK cells were infused at one of three doses (1 × 10^5^, 1 × 10^6^, or 1 × 10^7^ CAR-NK cells per kilogram of body weight) post lymphodepletion with fludarabine and cyclophosphamide	CRS, neurotoxicity, and GvHD were not observed, and levels of inflammatory cytokines (e.g., IL-6) did not increase over baseline; a response was achieved in 73% of patients; the infused CAR-NK cells expanded and persisted at low levels for at least 12 months	cord-blood NK cells engineered to express IL-15 and a CD19-targeting CAR showed long-term persistence and potent antitumor activity (2018^26^)
Allogeneic CD19-trageting CAR-NKT (2021^25^)	5 patients with relapsed or refractory B cell malignancies	CD19	co-expressed with shRNA targeting B2M and CD74	CAR-NKT cells were infused at escalating doses (1 × 10^7^ or 3 × 10^7^ cells/m^2^ of body surface area) post lymphodepletion with fludarabine and cyclophosphamide	grade 1 CRS was observed in 20% of patients; CR was achieved in 1 out of 2 ALL patients and 2 out of 7 NHL patients; the therapeutic cells expanded *in vivo* and peaked at 6 weeks, persisting through 12 weeks post infusion	CAR-NKT cells offered advantages over CAR-T cells, such as activating NK cells, priming tumor-specific CD8 T cells, and targeting CD1d-positive tumor via NKT TCR (2019^133^)

Drawing on lessons from autologous CAR-T cell therapy, allogeneic clinical trials typically employ lymphodepletion using fludarabine and cyclophosphamide, with some protocols also incorporating alemtuzumab. Fludarabine and cyclophosphamide are cytotoxic agents that reduce the number of circulating lymphocytes, including both T cells and regulatory immune cells. This temporary immune suppression creates a favorable environment, or "space," for the infused CAR-T cells to proliferate and function effectively.^143^^,^^144^ By weakening the host’s immune response, these agents help reduce pre-existing immunity that could interfere with CAR-T cell engraftment and persistence. Alemtuzumab, a monoclonal antibody that targets CD52, is also used in some regimens for additional immunosuppression. It depletes a wide range of immune cells, including T cells, B cells, NK cells, monocytes, and some dendritic cells.^145^ To avoid the depletion of allogeneic CAR-T cells, which would express CD52, these therapeutic cells are further engineered to knock out CD52, enabling them to avoid alemtuzumab-mediated clearance.^66^^,^^68^^,^^78^

In addition to these strategies, T cells can be engineered to resist specific drugs used in cancer therapy and transplant immunosuppression. For example, CAR-T cells have been modified for glucocorticoid resistance by disrupting the glucocorticoid receptor using ZFNs.^146^ Immunosuppressant-resistant universal CAR-T cells have also been developed by integrating CAR and mutated cyclosporine A genes into the *TRAC* locus, simultaneously disrupting TCR expression.^147^ Lentiviral transduction facilitates CAR expression, while TALEN-mediated edits target TRAC and deoxycytidine kinase, resulting in CAR-T cells that are resistant to purine nucleotide analogues.^148^ Furthermore, co-expression of human dihydrofolate reductase and inosine monophosphate dehydrogenase II conferred T cell resistance to the cytocidal and anti-proliferative effects of methotrexate.^149^ T cells can also resist rapamycin via a rapa-resistant mutant of mTor and can be directed to B lymphomas through CAR engineering expression.^150^ Additionally, a CRISPR-Cas9-based protocol can knockout the FK506-binding protein 12 gene, which is vital for tacrolimus’s immunosuppressive function.^151^ Such modifications enable therapeutic T cells to evade depletion and function effectively alongside immunosuppressive agents, enhancing tumor control, improving persistence, and reducing the risk of GvHD in allogeneic therapies.

In addition to lymphodepletion, several studies have used allogeneic CAR-T or CAR-NK cells from donors that are partially HLA matched with the recipient.^77^^,^^123^ Partial HLA matching helps balance immune compatibility while maintaining the practical benefits of allogeneic off-the-shelf therapies. HLA matching reduces the risk of GvHD, where donor immune cells attack the recipient’s tissues. By partially matching HLA molecules between the donor cells and the recipient, the likelihood of GvHD is minimized compared to fully mismatched cells. At the same time, partial matching decreases the chances of allorejection, where the host immune system recognizes the allogeneic cells as foreign and eliminates them.^77^^,^^123^ This strategy improves the persistence and efficacy of the infused CAR-T cells by enhancing their ability to engraft and expand within the patient.

Another key strategy to prevent host rejection involves knocking out HLA-I and HLA-II molecules, including critical genes such as *B2M*, *HLA-A*, *HLA-B*, *CIITA*, and *CD74* (Tables 2 and 3). The knockout of these genes helps reduce the recognition of the donor cells by the host’s T cells, which would otherwise target foreign HLA molecules and initiate rejection. This approach enhances the persistence of allogeneic CAR-engineered cells and improves therapeutic efficacy, allowing the engineered cells to evade the host immune system more effectively.

In clinical trials, these strategies have been applied to generate allogeneic CAR-engineered cells that demonstrate notable *in vivo* persistence. Some allogeneic CAR-engineered cells have shown significant expansion post infusion, peaking at around 6 weeks and persisting for up to 12 weeks.^25^ While challenges remain, including optimizing the balance between immune suppression and cell function, these findings highlight the potential of allogeneic cell therapies to provide effective, scalable, and widely accessible treatments for cancer.

### Therapy design

Recent clinical trials of allogeneic CAR-engineered cell therapy have employed various dosing strategies to optimize efficacy while minimizing allorejection risks (Tables 2 and 3). A key trend is the use of escalating dose approaches, allowing for adjustments based on individual patient responses and safety. For example, in a study treating B cell acute lymphoblastic leukemia (B-ALL), UCART19 cells were administered to adult patients at escalating doses from 6 × 10^6^ to 1.8–2.4 × 10^8^ cells per kilogram of body weight, showcasing a flexible dosing strategy.^66^ Similarly, ALLO-501 and ALLO-715 therapies also utilized escalating doses to treat B cell lymphoma and multiple myeloma, emphasizing dose modification to enhance outcomes while ensuring safety.^68^^,^^69^^,^^78^

Weight-based dosing is another key principle observed across various clinical trials, particularly important in pediatric populations where body size varies significantly. For instance, in the administration of UCART19 cells to children, doses ranged from 1.1 to 2.3 × 10^6^ cells/kg, underscoring the necessity of tailoring therapies to individual body weights.^66^ This personalized dosing strategy is designed to optimize the therapeutic efficacy while minimizing potential side effects associated with higher doses.

Additionally, infusion doses vary across different CAR-engineered cell types. CAR-NK and CAR-NKT cells were typically administered at lower doses than conventional CAR-T cells, with CAR-NK cells infused at doses ranging from 1 × 10^5^ to 1 × 10^7^ cells/kg.^25^^,^^123^ This conservative dosing approach likely stems from the limited patient cohorts and clinical trial data available, which restricts the ability to safely escalate doses for CAR-NKT and CAR-NK cells. In contrast, CAR-T cells were often given at higher doses, with some trials reporting doses as high as 9.0 × 10^8^ cells per patient.^82^ This disparity emphasizes the necessity to adapt dosing strategies based on the unique properties and therapeutic profiles of the various cell types.

Many trials incorporated combination therapies that included immunosuppressive agents, such as alemtuzumab, alongside standard lymphodepletion regimens of fludarabine and cyclophosphamide (Tables 2 and 3). This strategy is designed to establish an optimal microenvironment for the infused CAR-engineered cells while mitigating the risk of immune rejection. The approach often enabled higher total cell doses, balancing adequate immune suppression with the functionality of therapeutic cells.

Immune sensitization poses a significant obstacle in the clinical use of CAR-T cell therapies, as the immunogenicity of CAR components can trigger an immune response that results in the premature clearance of CAR-T cells, emphasizing the necessity for repeat dosing. To address this issue, some trials, such as ALLO-501A and CTX110, incorporate a redosing strategy to enhance allogeneic CAR-T cell expansion in patients.^69^^,^^72^ In the ALLO-501A trial, a second, consolidation dose of therapeutic cells, combined with lymphodepletion, provided additional benefits, with a complete response rate of 66.7%.^69^ Similarly, in the CTX110 trial, a second infusion led to CAR-T cell expansion in all patients and a deeper clinical response in 62.5%.^72^ Notably, the second CTX110 dose was well tolerated and demonstrated further clinical benefits.^72^

By utilizing escalating doses, weight-based adjustments, combination therapies, and redosing strategies, clinical trials are adopting more personalized and effective therapeutic strategies. These methodologies not only enhance the potential of achieving positive clinical outcomes but also address crucial safety concerns, facilitating the development of more accessible and scalable immunotherapy treatments.

### Clinical outcome: Safety

Safety profiles of allogeneic CAR-engineered cell therapies exhibit both promising outcomes and persistent challenges that require careful management. CRS is a common adverse event, observed in varying levels across different therapies. For instance, UCART19 exhibited grade 1–2 CRS in 91% of patients, with 14% experiencing grade 3–4 CRS.^66^ While ALLO-501 demonstrated grade 1–2 CRS in 21.7% of patients, ALLO-501A showed no cases of CRS, highlighting the variability in safety profiles across different product types.^68^^,^^69^ In the case of allogeneic CD7-targeting CAR-T cells, 67% of WU-CART-0071001 patients experienced CRS, whereas patients receiving cells from HLA-matched or haploidentical donors had grades 1–2 CRS in 90% of cases and grades 3–4 in 10%.^70^^,^^75^

GvHD presents another significant safety concern. For UCART19, 10% of patients developed grade 1 skin GvHD, and 60% of patients receiving CD7-targeting CAR-T from HLA-matched or haploidentical donors reported grade 1–2 GvHD.^66^^,^^70^ However, products such as HLA-mismatched CAR-NK and CAR-NKT therapies reported no instances of GvHD, highlighting their potential as safer alternatives.^25^^,^^123^ Cytopenia remains a frequent concern, with high incidence rates reported: 50% in CTA101 (CD19/CD20 dual-targeting CAR-T), 82.6% in ALLO-501 (CD19-targeting CAR-T), and 100% in some allogeneic CD7-targeting CAR-T therapies.^24^^,^^68^^,^^70^ Additionally, infections, particularly grade 3 or higher, were observed in multiple studies, affecting 23.9% of patients treated with ALLO-501.^68^ Treatment-related mortality was also reported in certain cases, indicating a need for enhanced monitoring and management strategies.

The overall safety of allogeneic CAR-engineered cell therapies reflects a complex interplay of efficacy and adverse effects. More in-depth evaluation and optimization of safety measures are essential to mitigate risks while maximizing therapeutic benefits, ultimately improving patient outcomes in this evolving landscape.

### Clinical outcome: Efficacy

The efficacy of various allogeneic CAR-engineered cell products demonstrates significant potential in treating hematologic malignancies. Notable response rates have been achieved across multiple therapies, particularly within the CD19-targeting cohort. For instance, UCART19 reported an overall response rate (ORR) of 48%, with a median progression-free survival of 2.1 months.^66^^,^^71^ In contrast, the HLA-mismatched CD19-targeting CAR-NK product achieved a remarkable 73% response rate, with CAR-NK cells persisting at low levels for at least 12 months in all patients.^123^ Similarly, allogeneic CD19-targeting NKT cells displayed promising outcomes, achieving complete remission in 1 of 2 patients with acute lymphoblastic leukemia and 2 of 7 patients with non-Hodgkin lymphoma.^25^ Likewise, ALLO-501 achieved a maximum complete response lasting over 15 months, with a complete response rate of 50%.^68^ Regarding CD7-targeting CAR-T cells, WU-CART-0071001 demonstrated a response duration of 86 days, while cells from HLA-matched or haploidentical donors showed persistence in 83.3% of patients at 6 months post infusion.^70^^,^^75^

The persistence of CAR-engineered cells is another critical factor influencing efficacy. Most cell products in clinical trials demonstrated *in vivo* expansion after either the first or second infusion, with CAR-T cell levels peaking around 6 weeks post infusion (Tables 2 and 3).^25^^,^^69^^,^^77^ This persistence is crucial for sustained antitumor activity and for improving long-term outcomes. Moreover, the immune response associated with CAR-NK therapies is particularly noteworthy, as these therapies often avoid CRS and neurotoxicity, suggesting a more favorable safety profile while still maintaining efficacy.^123^

## Manufacturing considerations for allogeneic cell products

Allogeneic cell therapies offer a promising alternative to autologous treatments but still face manufacturing challenges. First, scaling up production to generate enough cells for multiple patients requires large expansion platforms like stirred tank bioreactors, which can introduce issues such as shear forces, unstable gas exchange, and non-physiological conditions, potentially compromising product quality.^152^ Therefore, procedures for developing, transporting, and preserving donor cells must be thoroughly researched and rigorously validated to ensure reliability and quality.^153^^,^^154^ Second, while using healthy donor cells reduces variability, donor-to-donor differences persist. This has caused debate about repeatedly sourcing cells from a single donor or establishing a master cell bank (MCB).^153^ iPSC-derived MCBs provide a limitless supply but take years to establish, supporting large-scale, on-demand manufacturing. Importantly, robust testing protocols for cell banks should be implemented to ensure safety, effective quality control, and validated efficacy.^153^ These standards must account for the specific characteristics of the cells, the production process, and their intended clinical use, ensuring consistency and compliance with quality and regulatory requirements.^153^^,^^154^

A major challenge in increasing the scalability of gene editing for allogeneic CAR-engineered cell therapies is safely removing multiple genes while minimizing off-target effects. Tools like ZFNs, TALENs, and CRISPR-Cas9 have advanced preclinical and clinical research, but their off-target activity remains a concern.^14^^,^^155^ To address this, improved gene-editing technologies, such as CRISPR hybrid RNA-DNA (chRDNA), have been developed to enhance precision and scalability, reducing the risk of chromosomal abnormalities.^155^ Recent clinical trials show promising results, with minimal GvHD and host rejection in gene-edited allogeneic CAR-T products, demonstrating their safety and potential for large-scale therapeutic applications.^38^

Although establishing a manufacturing facility for allogeneic therapy requires a significant initial investment, off-the-shelf CAR-T therapies hold substantial promise for cost savings compared to autologous treatments.^156^^,^^157^ By utilizing a single healthy donor to produce multiple cryopreserved batches, the manufacturing process becomes more efficient, leading to lower costs for healthcare institutions and patients.^156^ To further drive down costs, several innovative approaches are emerging. For example, although lentiviral vectors are currently effective, their production is both costly and time intensive.^158^ Non-viral gene delivery techniques, such as electroporation and lipid nanoparticles, offer a cost-effective alternative by eliminating the need for expensive Good Manufacturing Practice (GMP)-grade viral vectors.^158^^,^^159^^,^^160^ Additionally, enhancements in production processes, such as optimized media and self-stimulatory cells, can further reduce expenses related to labor and raw materials.^158^ With these advancements, the cost of producing CAR-T therapies was predicted to decrease by up to 75% over the next 5 years.^158^

## Conclusions

Allogeneic CAR-T cell therapy offers substantial benefits, notably the ability to treat more patients in a timely manner, despite additional technical challenges. Clinical trial experiences have highlighted the limitations associated with manufacturing and administering autologous CAR-T cell products. However, two significant challenges exist in allogeneic cell therapy: the risk of GvHD and allorejection. The risk of GvHD is primarily a safety concern, closely related to patient morbidity and mortality; severe GvHD can be life threatening.^18^^,^^161^^,^^162^ Consequently, various strategies have been implemented to mitigate GvHD, making it a primary consideration in the development of allogeneic cell therapies. In addition, host immune cell-mediated allorejection is a critical factor affecting the efficacy of allogeneic therapies. While not the primary concern compared to safety issues, it remains essential to address to ensure therapeutic effectiveness. Various strategies have been developed to enhance efficacy and mitigate allorejection, such as genetic modifications to reduce immunogenicity and the use of immune-suppression protocols (Figure 3). This review comprehensively examines all aspects related to allorejection of off-the-shelf cell therapies, including the immunological basis, current mitigation strategies, and insights from recent clinical trials.

Immune suppression is a critical and viable clinical strategy in allogeneic cell therapy. It is important to recognize that immune reactivity can be triggered by polymorphic proteins beyond the HLA molecules. For instance, KIRs, which are expressed on NK cells and some T cells, interact with HLA molecules and exhibit significant polymorphism, influencing immune regulation and potentially leading to immune reactivity.^163^ Additionally, MHC class I-related chain A (MICA), a highly polymorphic protein, is known to stimulate immune responses, particularly in the context of transplantation and cancer immunity.^164^ Although HLAs are well established as key determinants of immune compatibility, researchers must also consider the role of other polymorphic proteins, such as KIRs and MICA, which may contribute to immune complications in cell-based therapies.

One of the significant challenges in assessing strategies to evade allorejection in allogeneic cell therapy is the development of translational assays that can determine whether a specific genetic modification has a clinical impact. While several assays have been established to evaluate host T cell- and NK cell-mediated allorejection, detecting rejection mediated by innate immunity remains considerably more problematic. Moreover, these assays predominantly rely on preclinical models, including *in vitro* systems and humanized *in vivo* models, which may not fully capture the complexities of the human immune response (Figure 2). Therefore, clinical testing is crucial to thoroughly evaluate the immunogenicity and potential allorejection of off-the-shelf cell products. Ultimately, the gold standard for demonstrating a lack of rejection is the *in vivo* persistence of the infused cells as measured in clinical settings.

Many technologies have been developed to circumvent host T and NK cell-mediated allorejection; however, the role of antibody-mediated allorejection is often underappreciated in efforts to safeguard off-the-shelf cell therapies and ensure safety. For example, after recognizing foreign HLA molecules, alloantibodies excreted by B cells will mediate allorejection in several ways, including recruiting various immune cells such as macrophages and neutrophils to target graft cells. Recent studies have focused on strategies to shield cell therapies from host antibody-mediated immune responses. For instance, Peraro et al. utilized bacterial immunoevasins to create "shield" CAR-T cells, which successfully cleaved cytotoxic IgG, including anti-CAR antibodies found in patient samples, and maintained robust antitumor activity in the presence of anti-cell IgG *in vivo*.^165^ Additionally, Gravina et al. engineered CAR-T cells to overexpress the IgG receptor CD64, which provided protection against both HLA and non-HLA antibody-mediated cytotoxicity without impairing the cells’ overall efficacy.^61^

To advance the development of allogeneic cell therapies, it is imperative to create robust and predictive assays that can bridge the gap between preclinical studies and clinical outcomes. Enhancing the sensitivity and specificity of assays for innate immune-mediated rejection is essential, as innate immunity plays a critical role in the initial response to transplanted cells.^166^^,^^167^ Additionally, integrating comprehensive immune monitoring in clinical trials will provide valuable insights into the mechanisms of allorejection and the efficacy of strategies designed to mitigate it. Collaborative efforts between researchers, clinicians, and regulatory agencies will be vital in establishing standardized methodologies. Ultimately, ensuring the long-term persistence and functionality of allogeneic CAR-engineered cells in patients will confirm the success of these strategies and contribute to the overall efficacy and sustainability of this promising therapeutic approach.

In addition, several critical questions need to be explored and resolved to advance the development of allogeneic cell therapy. These include enhancing current strategies to prevent host recognition and rejection without compromising the function of CAR-engineered cells, and determining the optimal level of host immune suppression required to prevent allorejection while minimizing the risk of infections and other adverse effects. Developing truly universal donor CAR-engineered cells that are compatible with any recipient without eliciting an immune response is another key objective. Ensuring long-term persistence and sustained functionality of allogeneic CAR-engineered cells in the host environment, understanding the long-term effects and potential risks associated with extensive genetic modifications to prevent allorejection, and identifying combination therapies that can mitigate allorejection while enhancing antitumor efficacy are also paramount. Addressing these challenges will be crucial for unlocking the full potential of allogeneic cell therapies, ultimately transforming patient care and advancing the field of cell-based immunotherapy.

## Acknowledgments

This work was supported by a UCLA BSCRC Innovation Award (to L.Y.), and an Ablon Scholars Award (to L.Y.). Y.-R.L. is a postdoctoral fellow supported by a UCLA MIMG M. John Pickett Post-Doctoral Fellow Award, a CIRM-BSCRC Postdoctoral Fellowship, a UCLA Sydney Finegold Postdoctoral Award, and a UCLA Chancellor’s Award for Postdoctoral Research.

## Author contributions

Y.-R.L. and L.Y. designed the study. Y.-R.L., Y.F., S.N., Y.C., and Z.L. wrote the manuscript. All authors have read and agreed to the published version of the manuscript.

## Declaration of interests

L.Y. is a scientific advisor to AlzChem and Amberstone Biosciences, and a cofounder, stockholder, and advisory board member of Appia Bio. None of the declared companies contributed to or directed any of the research reported in this article.
